# New and Old Challenges in Pediatric Health Policies

**DOI:** 10.3390/children9081196

**Published:** 2022-08-09

**Authors:** Tonia Vassilakou

**Affiliations:** Department of Public Health Policy, School of Public Health, University of West Attica, Athens University Campus, 196 Alexandras Ave, GR-11521 Athens, Greece; tvasilakou@uniwa.gr

Pediatric Health Policies represent a complex context, which integrates various aspects of children’s health and well-being, along the continuum of fetal life, infancy, childhood and adolescence. Care provided at every stage affects health, development and well-being in later years. Each one of these periods exhibits specific characteristics and developmental milestones and is affected by various factors and risks [1]. The interactive processes that determine a child’s health and development, driven by biological, environmental, socioeconomic and behavioral factors, operate through crucial and sensitive periods [2]. In order to achieve the aim of global children’s health, all these parameters should be considered by the stakeholders, and coordination among all involved bodies should be achieved. To this end, this Special Issue “Pediatric Health Policy” in Children provides a multifaceted approach to current Pediatric Health Policies taking into account the complex interactions between various meaningful determinants. 

Most societies recognize childhood and adolescence as priority fields. The concept of children’s rights has been first pointed out in the United Nations (UN) Convention on the Rights of the Child (CRC), which is the principal children’s rights document; in the CRC four areas of children, human rights have been described: survival, development, protection and participation [3]. Since then, a large volume of scientific research has revealed the need for interventions in several fields and a plethora of actions have been undertaken. A broad conceptualization of human capital includes health and wellbeing, knowledge, and interpersonal and socio-emotional skills needed to fulfill individual and societal potential [4]; human capital originates before conception and extends throughout childhood, adolescence, and beyond [5]. Furthermore, the Sustainable Development Goals (SDGs) provide a pathway to the development of human capital, which is considered crucial for our society [6].

Neonatal and infant morbidity and mortality rates have considerably improved during the past two decades. The under-five years’ mortality rate (U5MR) is broadly recognized as one of the more sensitive indicators of socio-economic status and quality of life, and its improvement reflects advances in health care services, premature newborn health care, immunization programs and improvements in sanitation [7]; nevertheless, U5MR remains unequally distributed across countries and regions, with the highest mortality rate being reported in West and Central Africa [8]. In 2020, an estimated 5 million children under the age of 5 years died, mostly from preventable by well-known and affordable interventions and treatable causes, such as lower-respiratory infections, diarrhea, malaria, injuries, measles, congenital anomalies, and tuberculosis; approximately half of those deaths, 2.4 million, occurred among newborns [9]. 

Poverty, low levels of maternal education, and poor health care are underlying determinants of most under-five deaths, along with known indicators, such as low birth weight (LBW) [10]. In this context, this Special Issue of *Children* brings to the foreground the humanitarian crisis in Yemen, as Coulibaly-Zerbo et al. present the adaptations in the nutrition program, in order to maintain the delivery of essential nutrition services to children under the age of five, providing a case study for other countries worldwide that might face similar challenges [11]. Moreover, in this Special Issue, Rutter critically evaluates the underlying concepts in the application of the adverse childhood experiences international questionnaire (ACE-IQ) as a policy tool, highlighting that the tool successfully covers school attendance and parental supervision, which mainly pertain to Western concerns, whereas global concerns, namely forced economic migration and famine, are not taken into account [12]. Huang et al. also focus on early life mortality, analyzing risk factors for Sudden Infant Death Syndrome (SIDS) in a case–control study on 953 SIDS cases and 1:10 matched controls, showing that the odds of SIDS were higher among offspring of younger mothers (aged less than 20 years) and infants in the eastern region of Taiwan [13].

Children who experience adversities in their development may not be able to fulfill their developmental potential. Policies should guarantee the right of every child to a healthy beginning of their life. This should begin during the preconception and pregnancy period when maternal lifestyle (nutrition, physical activity, stress, smoking, alcohol consumption, etc.) plays a crucial role. The window of the first 1000 days in a person’s life, from conception until the second year, is critical for future health and the formation of human capital [14]. Existing policies are mainly focusing in areas, such as maternal lifestyle during pregnancy, delivery method, prompt initiation of breastfeeding after delivery, exclusive breastfeeding at least for the first six months of life and proper weaning and transition to solid foods, which are important determinants of short and long-term health. Policies aiming at the healthy beginning of life have been widely adopted; in that context, several national strategies and action plans have been developed and implemented, incorporating childhood nutritional guidelines, and introducing new perspectives [15].

Childhood malnutrition, including undernutrition (wasting, stunting and underweight), micronutrient deficiencies, as well as overweight and obesity, is a triple burden and is one of the leading causes of poor health and a major impediment to personal development and achievement of full human potential worldwide [16]. According to a recent report, in 2019, 144 million children under 5 were stunted, 47 million wasted and 38.3 million overweight [17]. Childhood undernutrition may result in long-term effects that are irreversible, including impaired physical growth and cognitive development [16]. Poor nutritional status, especially among under-five children, is strongly correlated to vulnerability to diseases, delayed physical and cognitive development, as well as increased risk of morbidity and mortality [18]. Rates of childhood overweight and obesity are rising, both in high-income and low- and middle-income countries, and are associated with the risk of non-communicable diseases (NCDs), including diabetes mellitus, cardiovascular diseases mental health issues and various cancer types [19]. Policies for the prevention and management of malnutrition globally are essential for the healthy development of children. According to the fifth global Sexual, reproductive, maternal, newborn, child and adolescent health (SRMNCAH) policy survey, which covered 16 national policy areas, 75% of the countries have developed policies or guidelines concerning malnutrition; however, only 59% of them reported policies aiming to overweight and obesity [20,21]. Moreover, the report highlights the discrepancy between policies, interventions and achievements, and the need for the development of appropriate monitoring systems; in that context the need for further implementation and evaluation of the existing and future policies should be emphasised [21].

In this Special Issue of *Children*, Denburg et al. investigate the moral foundations of child health and social policies adopting a social constructivist lens in a critical interpretive synthesis of the literature, analyzing a total of 123 relevant articles. The authors identified three core values in child health policy, namely “potential, rights, and risk”. A narrow definition of children’s well-being is considered “as the absence of abuse, neglect, exploitation”, which may be measured by the response to their needs and satisfactory quality of life. Ideas about children’s well-being and best interests (BIC) incorporate a wide range of themes such as “predictive genetic tests, sexuality and sexual health, child welfare, public health, and research involving children” in Health and Social Policies. The authors concluded that most societies envisage children as “potent, vulnerable, entitled, and embedded”; the importance allocated to each one of these elements is crucial for the form of Child Health and Social Policies, as well as the consequent reactions of the society [22]. Examining current trends in the scientific and research community, Groff et al. performed a citation analysis of pediatric and adult randomized controlled trials in general medical Journals for the 2005–2018 period and showed that, contrary to adult RCTs, the number of pediatric RCTs did not grow significantly along with time, whereas pediatric RCTs were less cited, findings that describe a gap disfavoring pediatric RCTs in high-impact general medical journals [23]. Cressman et al. performed a comparative case study design in Ontario and Manitoba, Canada, evaluating the challenges of Joined-Up Governance (JUG) in the aforementioned two Canadian jurisdictions and showed that even within the same country that there are meaningful differences in arrangements and outcomes [24].

In the era of the COVID-19 pandemic, new challenges have emerged in the field of Pediatric Health Policies. In this Special Issue, Pappalardo et al. examined telemedicine in pediatric infectious diseases; telemedicine offers many advantages including time and financial saving, enhancement of collaborations with specialists and ongoing updates, however further application in real-life practice is necessary [25]. Furthermore, tracing emerging trends during the COVID-19 pandemic, Bottari et al. evaluated imaging studies performed in children with injuries at the pediatric emergency department and showed a significant decrease in the proportion of negative imaging studies, potential highlighting a degree of imaging inappropriateness in the era before COVID-19 [26]. 

Contemplating the aforementioned ideas as a whole, children are a population group characterized by unique needs that should be addressed by Pediatric Health Policies that take into account the ever-changing nature of global conditions and crises and ensure equitable access to health benefits. As Gabriel Mistral (Nobel Prize, 1945) said, “Many things we need can wait. The child cannot wait. Now is the time his bones are being formed, his blood is being made, his mind is being developed. To him we cannot say tomorrow, his name is today”.
